# A short primer on lung stereology

**DOI:** 10.1186/s12931-021-01899-2

**Published:** 2021-11-27

**Authors:** Matthias Ochs, Julia Schipke

**Affiliations:** 1 Institute of Functional Anatomy, Charité-Universitätsmedizin Berlin, Corporate Member of Freie Universität Berlin and Humboldt Universität zu Berlin, Philippstr. 11, 10115 Berlin, Germany; 2 German Center for Lung Research (DZL), Berlin, Germany; 3 Institute of Functional and Applied Anatomy, Hannover Medical School, Hannover, Germany; 4 Biomedical Research in Endstage and Obstructive Lung Disease, Member of the German Center for Lung Research (DZL), Hannover, Germany

**Keywords:** Alveoli, Dimension trap, Disector, Histology, Lung, Microscopy, Morphometry, Reference trap, Sampling, Stereology

## Abstract

The intention of this short primer is to raise your appetite for proper quantitative assessment of lung micro-structure. The method of choice for obtaining such data is stereology. Rooted in stochastic geometry, stereology provides simple and efficient tools to obtain quantitative three-dimensional information based on measurements on nearly two-dimensional microscopic sections. In this primer, the basic concepts of stereology and its application to the lung are introduced step by step along the workflow of a stereological study. The integration of stereology in your laboratory work will help to improve its quality. In a broader context, stereology may also be seen as a contribution to good scientific practice.

## Background

Your nice and elaborate experimental study demonstrating that your favorite molecule is essential in the pathogenesis of a common lung disease is ready for submission. You chose a very prestigious journal. After a few weeks, you receive the reviewers’ comments. One (or more) of the reviewers is asking for a formal quantification of the histological findings in your animal model. What now? After a brief search, you find out that there exists something called stereology (in case the reviewer didn’t already mention it) that is considered the gold standard to quantitate lung architecture in microscopy. There is even an official research policy statement by the American Thoracic Society (ATS) and the European Respiratory Society (ERS) about this [20]. You approach one of the leading scientists in the field of lung stereology and ask for help. After a few emails have been exchanged, this expert tells you that your study cannot be rescued at this stage, because all you have available for analysis is a few paraffin sections taken arbitrarily from each mouse lung without knowledge of the volume of the fixed lung, but these alone are not suitable for stereological analysis (compare Table 1 for common problems). Sadly, without these data the journal is not willing to accept your paper for publication.

**Table 1:** Most common problems in micro-structural assessment of the lung

Step	Problem	Comment/solution
Reference space	Lung volume not measured	Avoid “reference trap” by measuring lung volume and reporting data related to the whole lung
Sampling	Biased by taking tissue samples and fields of view either preferentially (e.g. “most interesting” areas) or always at the same site (e.g. “mid-sagittal section along the main bronchus”)	Use appropriate unbiased sampling protocols (e.g. SURS in combination with fractionator sampling) which gives each part of the lung an equal chance for being analyzed
Orientation	Not taken into consideration	Surface area and length estimation of anisotropic structures require randomization of orientation in space (IUR samples)
Embedding	Shrinkage in paraffin	Avoid shrinkage by using appropriate embedding protocols (e.g. glycol methacrylate after osmication)
Number estimation	Particles counted as profiles on single thin sections	Avoid “dimension trap” by using the disector for unbiased estimation of particle number

Modified from [35]

*SURS* systematic uniform random sampling, *IUR* isotropic uniform random

What went wrong? If you don’t know, this primer is for you. It aims to help you to do better next time, in particular by planning ahead for a quantitative assessment of lung micro-structure. Don’t be deterred by the mathematical theory of stereology (although we have to confess that some of the original articles we reference in this primer are a tough read). Rest assured that this is actually an advantage because it provides a solid scientific foundation (from stochastic geometry) for what you will be doing. Think of stereology as a toolbox. These tools have names that may sound unfamiliar to you. Therefore, you find a little glossary that defines common stereological terms in Table 2. The basic principles how to use stereology can be learnt in a few hours. The production of data is straightforward. For (almost) every task there is an appropriate tool. You just have to be willing to change a few lab habits regarding the processing of lung tissue for “standard histology”. This is probably the biggest hurdle. Then the rest is easy.

**Table 2:** Glossary of stereological terms

Stereological term	Definition
Bias	Systematic error. A systematic deviation of the average estimate from the true value. Absence of bias is termed accuracy
Cavalieri estimator	An unbiased method for estimating the volume of an object by slicing it in parallel sections of a known thickness and estimating the area of the cut surfaces with a point grid. The volume is calculated by multiplying the total surface area of all sections with the section thickness
CE	Coefficient of error. A mathematical expression of the imprecision (variance) of an estimate. Can be predicted to adjust the sampling effort in relation to the overall coefficient of variation (CV) of a study group
Disector	A 3D stereological test system (test volume) for sampling and counting objects. By either using two sections (physical disector) or by focussing through one thick section in z direction (optical disector), a known z distance with a defined counting area is sampled, and particle number in 3D space can be estimated
Fractionator	An unbiased sampling design that is based on keeping track of sampling fractions at each subsampling step to obtain total values by multiplying the counted objects at the final sampling step with the inverse of the sampling fraction. The method can optimally be combined with the optical disector (optical fractionator)
Isector	A method for generating IUR sections by embedding the tissue in spherical molds to randomize orientation in further tissue processing
IUR	Isotropic uniform random. An unbiased sampling design that randomizes for spatial orientation of microscopic sections (IUR sections). Tools to generate IUR sections are the isector or the orientator
Orientator	A method for generating IUR sections via a two-step process, including randomized dis-orientation along two different axes
Reference volume	The space from which samples are taken and in which particular stereological measurements are performed, e.g. total lung volume. Knowledge of the reference volume is crucial to convert densities estimated with stereological test systems to total quantities
SURS	Systematic uniform random sampling. An unbiased sampling design that randomizes for location (e.g. the selection of tissue blocks or fields of view). Includes a systematic and a random component, by selecting the first position at random and the selection of all other positions with a constant sampling interval
VUR	Vertical uniform random. An unbiased sampling design for randomization of spatial orientation. Combines randomization of microscopic sections in two dimensions (VUR sections), thus maintaining a specific horizontal plane, with a sine-weighted curved test line orientation (cycloids) to randomize the interaction with test lines in the third dimension

Modified from [22]. See also the glossary in [32]

In this primer, we focus on the application of stereology in combination with conventional microscopic techniques (e.g. basic light microscopy). In principle, stereology can be combined with any microscopy (actually any imaging) method (see [22]). The point we want to make here is that no fancy extra equipment is needed to do proper basic lung stereology (which also means that no excuses are accepted). It is all about the design of the study and the appropriateness of the methods for your specific needs.

## Doing lung stereology step by step

Let’s go through the workflow of a typical stereological study, from its planning stage to the final publication. It consists of 7 crucial steps. Along the way we will familiarize you with the basic concepts of lung stereology and the important points to consider (Table 3). Cross references between these steps are given to show how they are interrelated. We will also provide references for further reading.

**Table 3:** The 7 crucial steps of a stereological study of the lung

Step	Points to consider
1. Planning your stereological study	Pilot study Target compartment Stereological parameters Sampling and analysis design
2. Preparation of lung tissue	Route of fixation Composition of fixative Post-fixation and processing Dehydration and embedding
3. Definition and measurement of the reference space	Fixed lung volume: -Archimedes principle -Cavalieri estimator
4. Sampling	Randomization of location: -Systematic uniform random sampling -Cascade sampling -Stratified sampling -Fractionator sampling Randomization of spatial orientation: -Isotropic uniform random sections -Vertical sections
5. Doing the measurements	Dimension of stereological parameter of interest Dimension of geometric probe (test system) Disector for number estimation
6. How many counts are enough?	Accuracy Precision Coefficient of error
7. Reporting your methods and results	Total values Ratios and inverse of ratios Scatter plots Standard deviation

This table summarizes the crucial steps involved in a typical stereological study of the lung and the way this primer is organized. Points to consider at each of these 7 steps are covered in the text. Stereological terms are explained in Table 2

### Step 1: Planning your stereological study

**Fig. 1:**
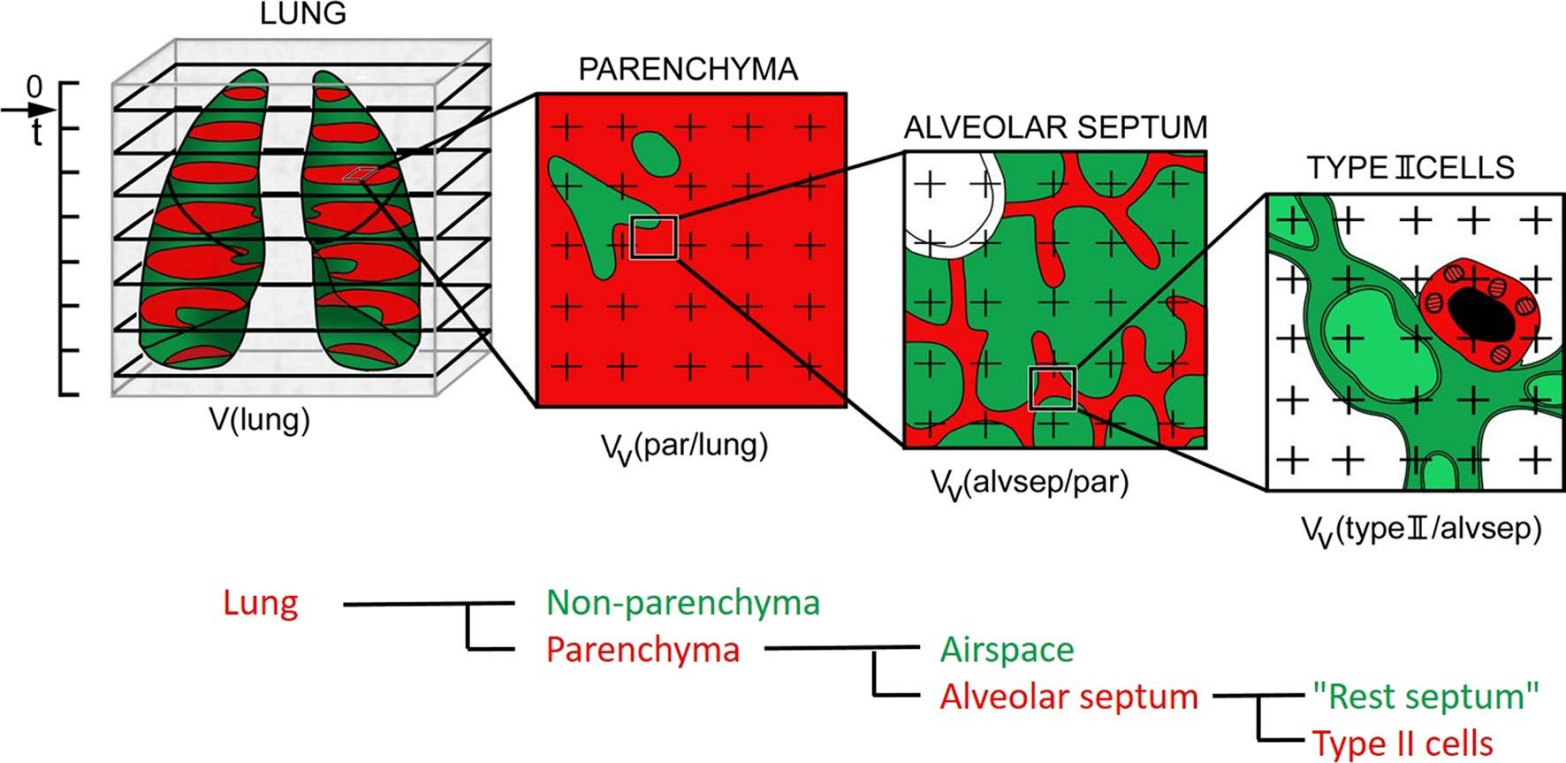
Illustration of a *cascade* *sampling* *design* in the lung, using the estimation of the total volume of alveolar type II cells in a lung as an example. At each level, from macroscopic via light microscopic to electron microscopic, the principles of unbiased sampling, e.g. by systematic uniform random sampling, have to be applied (Step 4). At the macroscopic level, the lung is cut completely into horizontal slices of thickness t, starting at a random position between 0 and t (arrow). These slices serve two purposes: They can be used for the estimation of total lung volume by the *Cavalieri* *estimator* (Step 3) as well as for sampling of tissue blocks for light and electron microscopy (see Fig. 3). Total lung volume, V(lung), is the product of t and the total cut area of the apical side of all slices (shown in red). At a low light microscopic magnification, the volume fraction of parenchyma within lung, V_V_(par/lung), is estimated by point counting. The volume fraction of alveolar septum within parenchyma, V_V_(alvsep/par), is estimated by point counting at a medium light microscopic magnification (compare Fig. 5). The volume fraction of type II cells within alveolar septum, V_V_(typeII/alvsep), is estimated by point counting at a medium electron microscopic magnification. The total volume of type II cells, V(typeII), is then obtained as V(typeII) = V(lung) · V_V_(par/lung) · V_V_(alvsep/par) · V_V_(typeII/alvsep). Note that in this cascade sampling design, the phase of interest at one level (red) becomes the reference phase (red + green) at the next level. Modified after [34]

You should have the concept of your stereological design ready before the practical part of your study starts. Most likely you will have to do a little *pilot* *study* in advance. This will give you an impression of the changes in lung structure in your model. Do a thorough qualitative microscopic analysis. What exactly are the alterations in lung structure between your experimental groups that you want to quantitate? Think of the lung as being composed of different compartments which can be subdivided further (which may require a cascade of increasing magnification levels, see Step 4 below and Fig. 1). What is your target compartment? Whatever it is, always derive it from the whole lung, and also relate your measurements in that target compartment back to the whole lung when reporting your final data (see Steps 3 and 6 below).

Express the alterations in your target compartment as changes in *stereological* *parameters* such as volume (V), surface area (S), length (L) or number (N). Derived from these parameters, there are others like mean particle volume ($\overline{\nu }$ = V/N) or mean barrier thickness ($\overline{\tau }$ = V/S). Note that these parameters differ in their dimension: V has dimension 3, S has dimension 2, L has dimension 1, and N has dimension 0 (we’ll come back to that in Step 5 below). Accordingly, $\overline{\nu }$ has dimension 3 and $\overline{\tau }$ has dimension 1. Note that most likely you will need a combination of several parameters to characterize structural changes in the lung. For example, an emphysema-like phenotype can be quantitated by changes in total alveolar surface area (decrease in S), total alveolar number (decrease in N) and mean alveolar volume (increase in $\overline{\nu }$). More details on appropriate stereological parameters for various animal models of human lung disease are given in [30].

Your small pilot study serves another purpose: It can be used to determine your *sampling* *and* *analysis* *design* for your definite study. This includes e.g. the number of individuals per group, the number of tissue blocks per lung, the number of sections per tissue block, the number of fields of view per section at a given magnification, and the stereological test system (see Step 6 below).

In case the total alveolar surface area of the lung is of interest in your study, how can you measure this? Obviously not directly. Neither can you roll out the inner surface of a lung on a lab bench and use a ruler nor can you trace the alveolar surface in microscopic sections for that purpose. Structural measurements in microscopy are a challenge for two reasons (and forgetting about these challenges leads to common mistakes, see Table 1):

1. There is a *reduction* *in* *size*. The thin microscopic sections you investigate constitute only a fraction, actually an infinitesimal sample, of the whole lung. However, you want to know the total alveolar surface area of that lung. So you have to measure surface density (the ratio of surface per unit volume) in these sampled sections first and then multiply this surface density by the reference space (total volume of lung parenchyma) to calculate total alveolar surface area in the lung (only then the units make sense—check yourself). Therefore, think of the total volume of the (fixed) lung (see Step 3 below) as the starting point for taking your samples (see Step 4 below) as well as the end point for reporting your data (see Step 6 below). So, measurements on microscopic sections are not the end point, because they are ratios, not totals. These ratios are subject to the “*reference* *trap*” [5] where a change in the ratio can be due to a change in the numerator (alveolar surface area) or the denominator (parenchymal volume) or both, thus leading to ambiguity in interpretation. Don’t fall into this “reference trap” by reporting ratios. Instead, measure the reference space and compute total values (see Steps 3 and 6 below).
2. There is a *reduction* *in* *dimension*. The microscopic sections you investigate are thin slices. Although the structures you are interested in are three-dimensional (3D) entities, their representation in sections is two-dimensional (2D). Something that looks small in your section, e.g. an alveolar profile, might be much bigger but is cut at the periphery. A line in your section could represent a tubular as well as a plate-like structure. Depending on the section plane, a tubular structure could also appear as a point in your section created by a transect. The alveolar surface you are interested in (2D) will appear as a thin boundary line (1D) towards the alveolar lumen. In short, we lose one dimension due to the sectioning process, and therefore we lose important qualitative and quantitative information. In analogy to the “reference trap”, this has been termed the *“dimension* *trap”* [22]. The appearance of 3D structures in nearly 2D microscopic sections does not represent the full reality. Don’t fall into this “dimension trap” by using particle profile counts in sections to obtain particle number in 3D space. Instead, use the disector (see Step 5 below).

What is the basic idea of stereology? To address and overcome these two challenges. What is needed are proper ways of sampling (such that the tissue which is selected for microscopic analysis is truely representative of—and thereby can be related to—the whole lung) and measurement (such that true 3D data are obtained although only nearly 2D microscopic sections are available for analysis). This is exactly what stereology offers: methods, which are derived from stochastic geometry, to obtain quantitative structural information of irregular objects from microscopic (or any imaging) datasets which are based on measurements on properly sampled (physical or virtual) sections. A fundamental characteristic of these methods is the fact that they do not rely on any assumptions regarding the shape, size, spatial distribution or orientation of the objects. Therefore, stereological methods are *unbiased* *by* *design* (*design-based*), in contrast to other methods which use such geometric model assumptions (model-based). This is why stereology is the method of choice to obtain quantitative structural (morphometric) data in microscopy and became the current standard in lung research [20]. An example of an algorithm for planning a stereological study is illustrated in [37].

### Step 2: Preparation of lung tissue

This part is of outmost importance, but often underestimated or ignored. The well-known rule *“garbage* *in,* *garbage* *out”* applies here. Even with stereological methods that are unbiased by design, you may produce severely biased data if the lung samples have undergone tissue deformation (e.g. shrinkage during dehydration and embedding).

Unfortunately, there is no gold standard for lung fixation. However, a few conditional silver standards have been defined [20]. Note that whenever you fix a lung, you produce an artifact—but you should do so in a controlled, consistent and reproducible manner. Points to consider are e.g. the route of fixation (instillation via the airways versus perfusion via the vasculature), the composition of the fixative (which preferably should contain glutaraldehyde), post-fixation and processing (which should include osmium tetroxide, even for light microscopy), dehydration and embedding (which should avoid paraffin and employ glycol methacrylate instead). We have evaluated the effects of different protocols with regard to changes in mouse lung tissue dimensions [42]. Briefly, the area of slices from fixed mouse lungs shrinks to about 60% when embedded in paraffin and to about 70% when embedded in glycol methacrylate according to the standard instructions of the manufacturer. Only when these slices are postfixed in osmium tetroxide before embedding in glycol methacrylate, the area remains almost constant at around 97%. Note that assuming equal shrinkage in all experimental groups is not an option—there are examples in the scientific literature where this assumption turned out wrong and led to false conclusions. You will only be able to obtain biologically meaningful values, when you faithfully preserve tissue dimensions. Don’t waste your time analyzing garbage. For more information on processing and embedding of lung samples for stereology, see [31].

### Step 3: Definition and measurement of the reference space

**Fig. 2:**
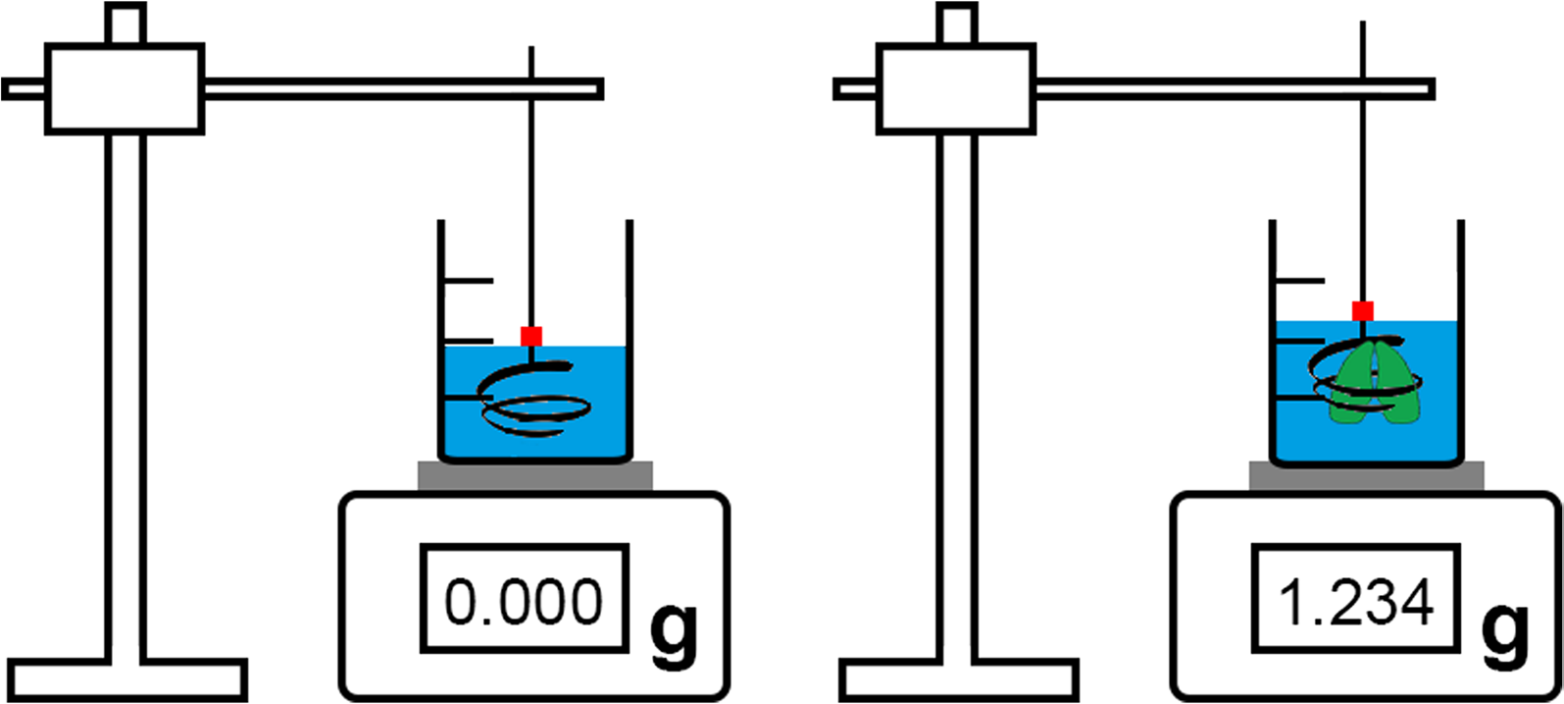
Estimation of the volume of the fixed lung (Step 3) according to the *Archimedes* *principle*. Based on buoyancy, the weight of the displaced fluid (e.g. saline) is measured. Since the fixed lung is lighter than the fluid, it tends to emerge and thus has to be submerged by a coiled wire connected to a lab stand. The lung and wire must not touch the wall of the beaker. The wire has a mark to adjust it to the fluid level before and during measurements to make sure it does not contribute to the measured volume. The volume of the lung (in mL or cm^3^) is shown as weight gain (in g)

**Fig. 3:**
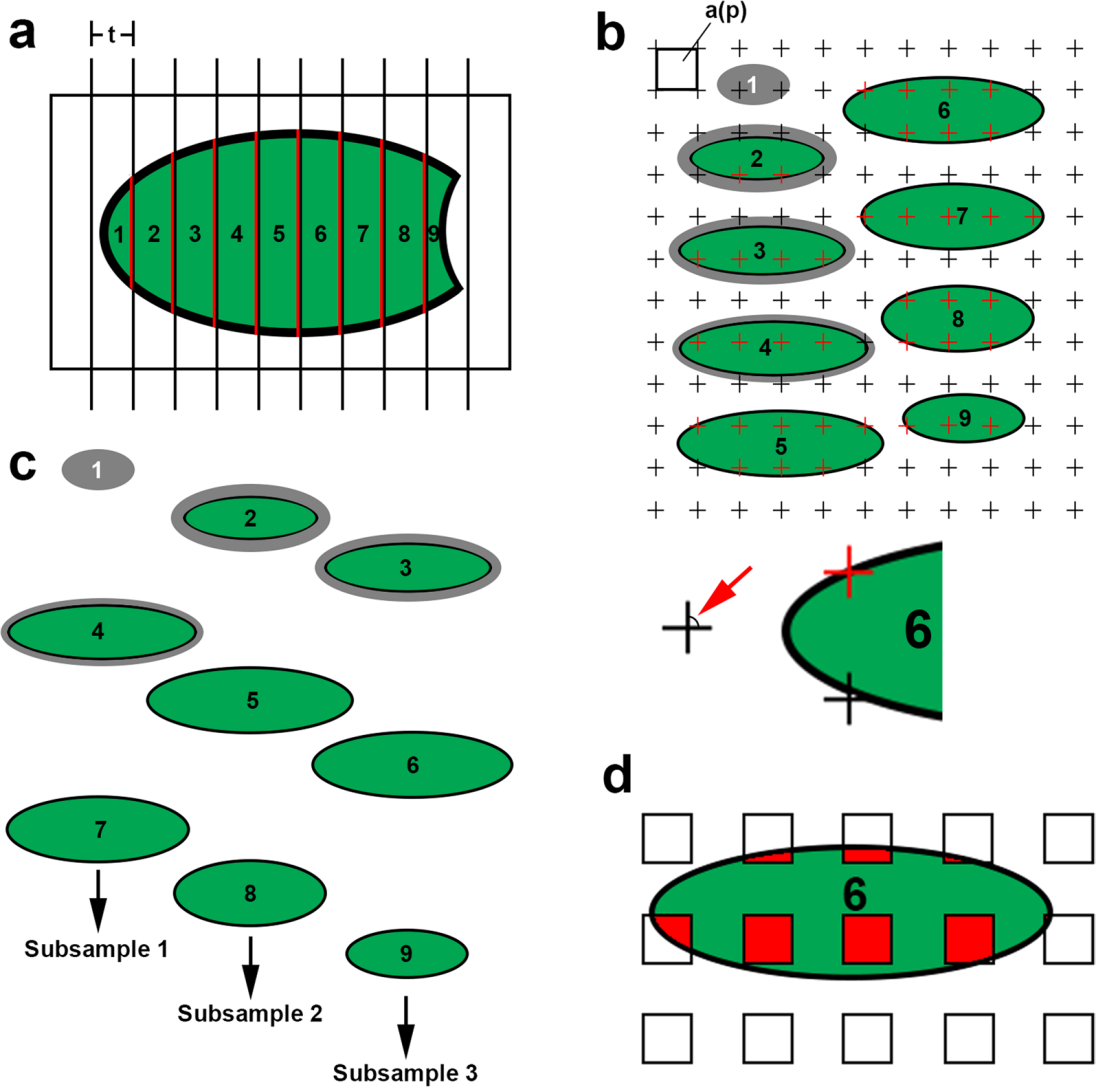
Estimation of the volume of the fixed lung (Step 3) according to the *Cavalieri* *estimator* and subsequent sampling. **a** The lung is embedded in agar and cut into slices of equal thickness t, with a random start in the agar outside the lung, thus following the principles of *systematic* *uniform* *random* *sampling* (*SURS*). Slice thickness t is adjusted to obtain around 8–10 slices. **b** The slices are placed on a tray with a consistent orientation, e.g. all apical surfaces upwards. The total volume of the lung, V, is calculated as the sum of the cut surface areas, A, of all slices (indicated in red in a) multiplied by the slice thickness t: *V* = *ΣA****·****t.* The cut surface areas, A, are estimated by counting points, P, hitting the cut surface areas using a grid with a known area per point, a(p), as: *V* = *ΣP****·****a(p)****·****t.* A constant definition for a point “hit”, P, is needed, e.g. the upper right corner of the crosses that symbolize the test points (indicated by red arrow). Based on this definition, correct point counts are shown in red. Note that in this example, where the slices are oriented with their apical side upwards, the first slice does not contain an apical cut surface and thus does not contribute any point counts. Accordingly, the uncut pleura at the lateral sides, here visible on the first four slices when viewed from above (grey), is also excluded (black test points) whereas the cut pleura at the apical surfaces (black) is included (red test points). **c** This collection of fixed lung slices can also be used as the starting point for *SURS* (Step 4). For example, if 3 different embedding protocols shall be used in the study (e.g. embedding in glycol methacrylate for light microscopic stereology, embedding in paraffin for immunohistochemistry and embedding of smaller subsamples in epon for electron microscopy), the slices are randomly assigned to these 3 protocols with a constant sampling interval (here 3). Thus, slices 1, 4 and 7 will be embedded according to protocol 1, slices 2, 5 and 8 will be embedded according to protocol 2 and slices 3, 6 and 9 will be embedded according to protocol 3. Note that all slices have to be sampled for embedding, even those who had to be excluded from the Cavalieri estimator (here slice 1). Also note that each of these 3 subsamples obtained by SURS constitutes a fraction of 1/3 of the whole lung. They can therefore also be used as starting point for *fractionator* *sampling*. **d** The principle of SURS consists of a random component (the selection of the first sample) and a systematic uniform component (the constant sampling interval for the selection of all other samples). SURS also applies to the subsequent stages of the sampling sequence, such as the selection of smaller subsamples (e.g. for electron microscopy) or the selection of fields of view on histologic sections from an embedded lung slice. The sampling interval is adjusted to achieve the desired number of samples

For your stereological study, you will need a biologically meaningful reference space that you have to measure at the beginning. As the pioneer of design-based biomedical stereology, Hans Jørgen Gundersen, once phrased it: *“Never* *ever* *not* *measure* *the* *reference* *space!”*. Use the fixed lung. Then you have two options: based on the *Archimedes* *principle* (buoyancy; you measure the weight of the fluid displaced by the immersed lung) [40] or based on the *Cavalieri* *estimator* (you measure the cut surface areas of the sliced lung by point counting and multiply these by the thickness of the slices) [28] (Figs. 2, 3). Details on lung volume measurement are given in [31] and [41]. A direct comparison of the two methods is provided by [49].

### Step 4: Sampling

Tightly interrelated with the measurement of the reference space and processing of lung tissue is the sampling of tissue blocks for microscopy. Actually, stereology is all about rigorous unbiased (and thus, truely representative equal opportunity) sampling. A bad sample will inevitably result in bad data and thus bad science. In general, sampling must be randomized for location, i.e. each part of the lung has the same chance for being selected for analysis. For anisotropic structures with a preferential orientation in space (e.g. major conducting airways), some parameters (e.g. surface area and length) also require randomization for spatial orientation.

Various stereological sampling designs have been developed. The basic principle for randomization of location is *systematic* *uniform* *random* *sampling* (*SURS*). The first sample is chosen randomly but determines the position of all other samples which are selected by a predefined constant sampling interval (Fig. 3). Depending on the target compartment you are interested in, you may have to follow this SURS principle along a cascade of microscopic magnifications (e.g. distinguishing parenchyma vs. non-parenchyma at a low light microscopic magnification, but distinguishing alveolar septum, alveolar airspace and alveolar duct airspace within parenchyma at a higher light microscopic magnification). This principle, where the phase of interest at a lower magnification becomes the reference phase at a higher magnification, has been termed *cascade* *sampling* (Fig. 1). Along this cascade, you must not break the SURS chain at any point. Also keep in mind that the microscopic resolution influences the data. This is of particular relevance for alveolar surface area estimation where the well-known “coast of Britain effect” leads to higher values with higher resolution because finer irregularities become visible [39]. Selective targeting of subcompartments within the lung, which can either be defined by anatomy (e.g. individual lung lobes) or by pathology (e.g. lesioned regions), is addressed by *stratified* *sampling* where those subcompartments are sampled seperately. The basic concept of *fractionator* *sampling* [12, 13] is to keep track of the sampling fraction throughout the cascade of sampling stages (e.g. slices, slabs, blocks, sections, fields of view). Fractionator sampling can easily be combined with SURS (Fig. 3). Its great advantage is that particle number can be estimated without bias independent of tissue deformation by multiplying the total counts at the final sampling step by the inverse of the sampling fraction along all sampling stages.

Randomization of spatial orientation yields *isotropic* *uniform* *random* *(IUR)* sections. Tools for randomization of orientation and production of IUR sections are the *orientator* [23] and the *isector* [33]. For cases where the complete dis-orientation provided by these methods is inconvenient (e.g. for layered epithelia where a particular, e.g. horizontal, orientation of the basement membrane should be maintained), *vertical* *sections* in combination with a cycloid test system have been developed [1]. General concepts of sampling for stereology are introduced in [25], the efficiency of systematic sampling is discussed in [14] and in [18], and details on stereological sampling of lungs for location and orientation are given in [31].

### Step 5: Doing the measurements

**Fig. 4:**
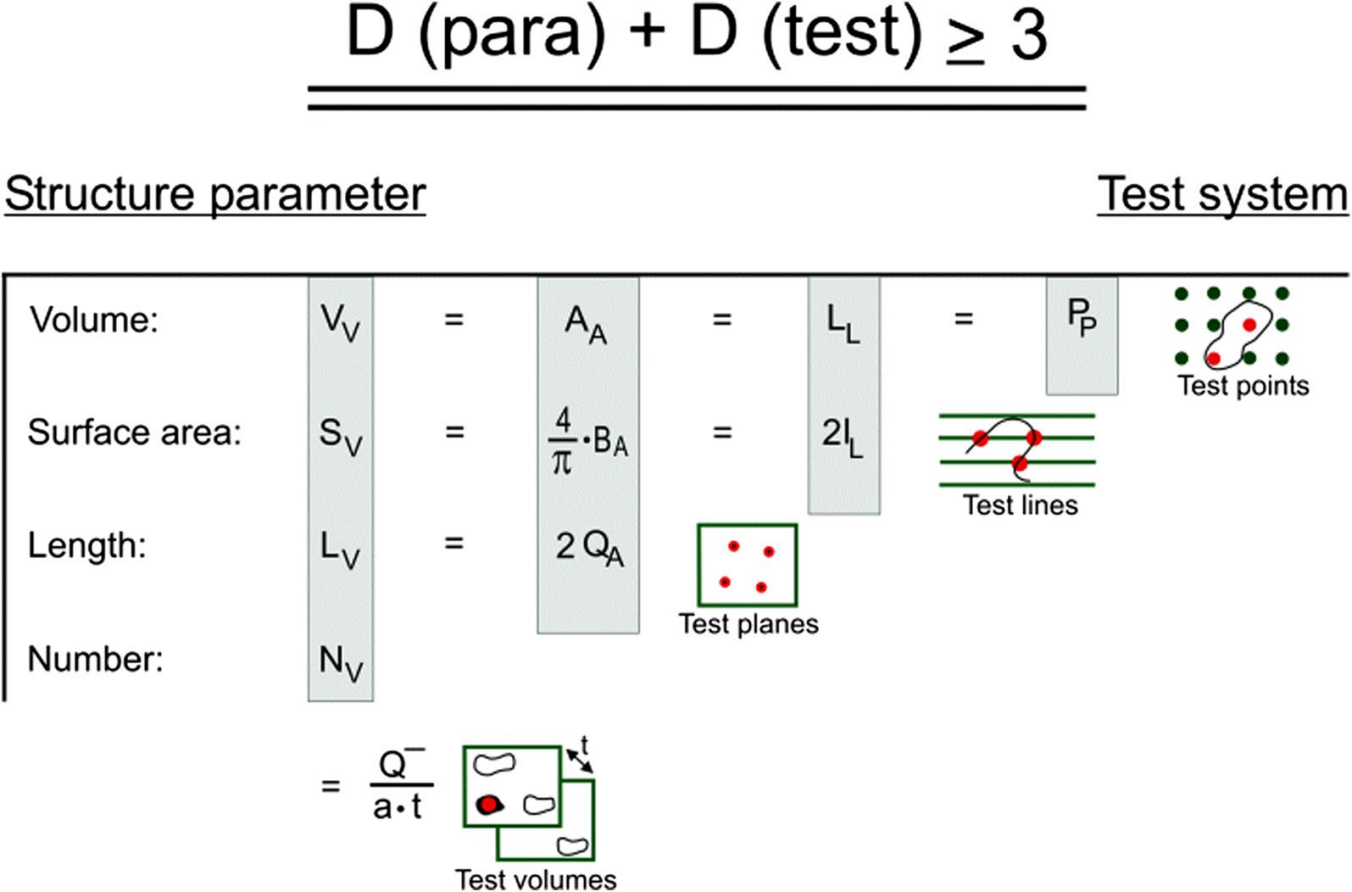
*Stereological* *test* *systems* and their relation to parameters of interest. The dimension of the parameter that is estimated plus the dimension of the test system containing geometric probes that is used to estimate it equals at least 3. Therefore, test points (dimension 0) “feel” volume (dimension 3), test lines (dimension 1) “feel” surface area (dimension 2), test planes (dimension 2) “feel” length (dimension 1), and only test volumes (dimension 3) “feel” number (dimension 0). In practice, test volumes are generated by using section pairs or thick sections (disectors) from one tissue block. For volume density estimation, test points are counted if they lie in the volume, i.e. hit the cut area in the section (red). For surface area density estimation, intersections are counted if the test line intersects the surface, i.e. hit the cut boundary line in the section (red). For length density estimation, transects are counted if the test plane transects the line, i.e. hits the line to create cut profiles (red). For number density estimation, particles are counted if their top lies in the test volume (disector), i.e. either they are present in one disector section but not the other (physical disector) (red), or their tops become visible while focussing through a thick section (optical disector). To avoid the “reference trap”, these estimates of densities (ratios) per unit reference volume have to be converted to total values by multiplying them with the total volume of the reference space (see Fig. 1). From [34]

**Fig. 5:**
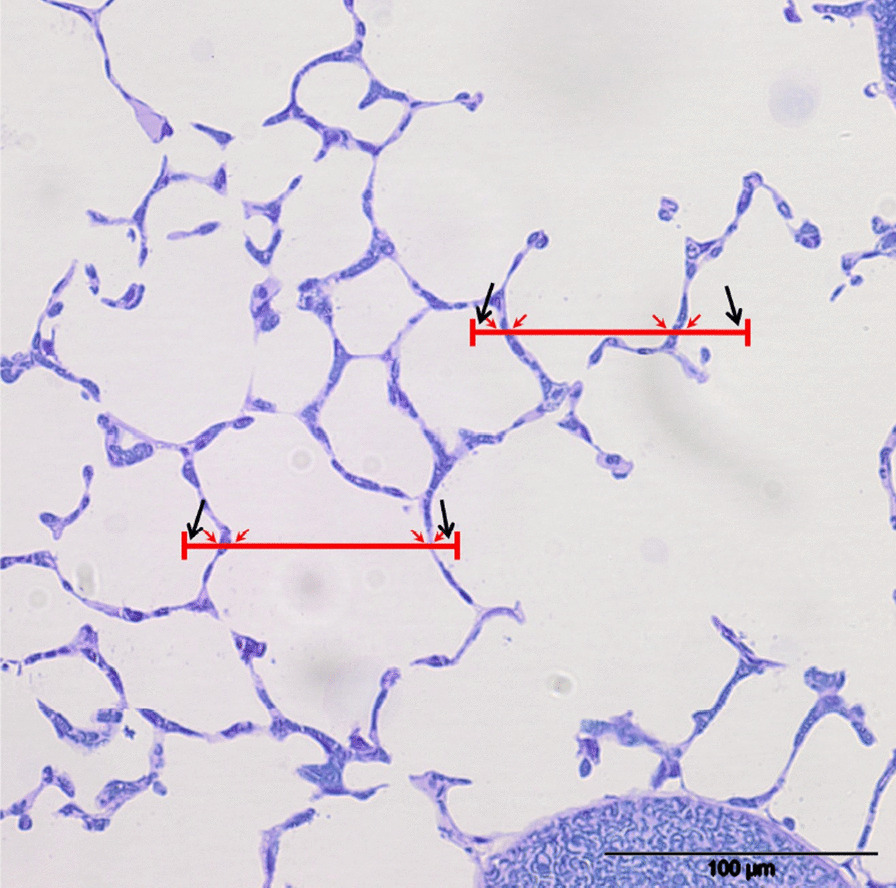
*Point* *and* *intersection* *counting* for estimation of volume and surface area. In case volume and surface area should be estimated on the same fields of view, test points and test lines can be combined into test line segments. These test line segments contain two test points, one on each end. Test point counts (large black arrows) may fall on alveolar airspace, alveolar duct airspace or alveolar septum, thus differentiating the parenchyma into compartment volume fractions (compare Fig. 1). Intersections of test lines with alveolar surface (small red arrows) are counted for surface area estimation. From Schneider and Ochs [14]

**Fig. 6:**
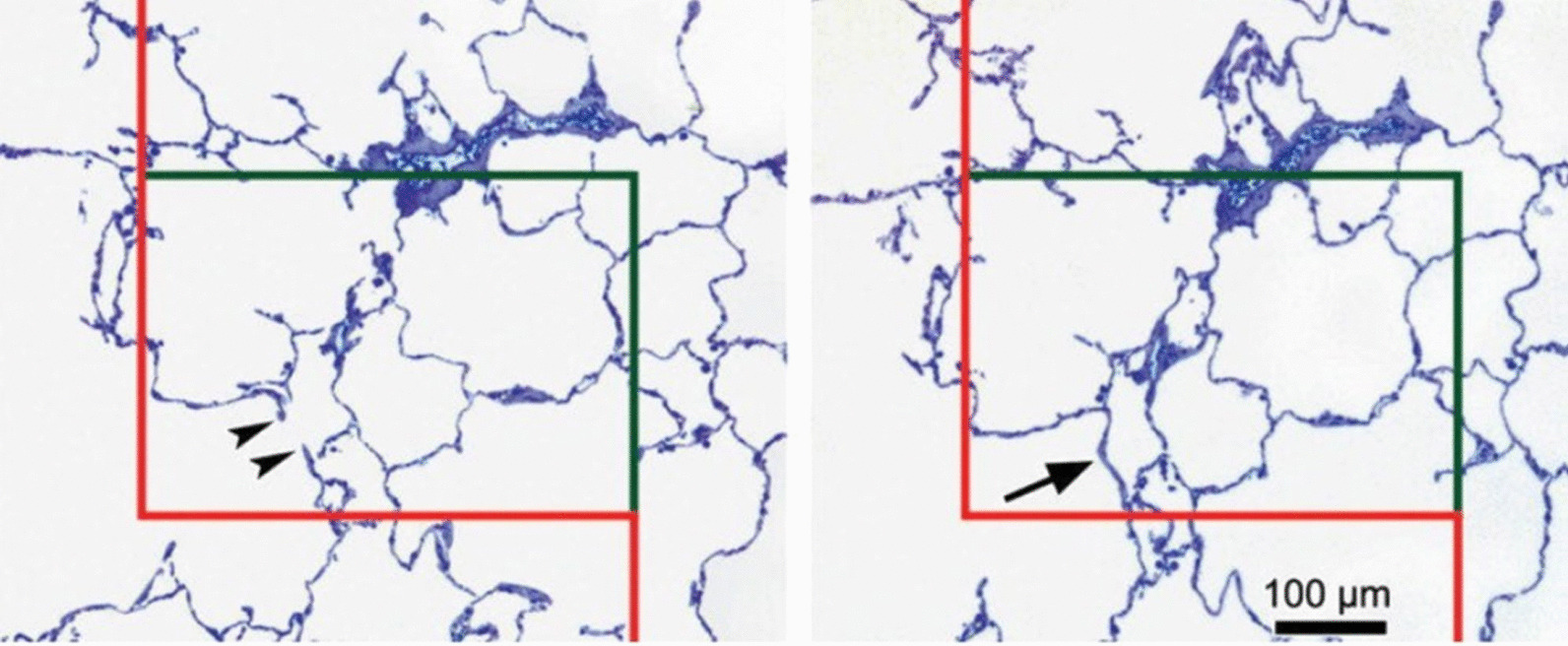
* Physical* *disector* for counting of alveolar openings to estimate alveolar number. Counting event is the presence of an alveolar opening in one section (left, indicated by arrowheads at the free septal edges that belong to the entrance ring) but not the other (right) where the alveolus is closed by a bridge (arrow). The disector volume is defined by the area of the unbiased counting frame [11] multiplied with the distance between the two sections (here 9 µm). The unbiased counting frame has inclusion lines (green) and exclusion lines with extensions (red). The counting rule allows disector counts of particles inside the counting frame, where they may touch the inclusion line, but not the exclusion line. Modified from [34]

Now it’s time to generate real data. There is good news: The “measurements” in stereology are very simple and therefore quick and efficient. They are actually reduced to simple counts (to quote Hans Jørgen Gundersen again: *“Simplicity* *is* *strength!”*). Test systems which are (digitally) overlaid over the fields of view interact with structures in the lung in such a way that counting events are created. These test systems consist of geometric probes which have a dimension suited for the parameter of interest (see Figs. 4, 5, 6; Table 4). The dimension of this parameter of interest plus the dimension of the geometric probe has to equal at least 3. Thereby, stereology provides real 3D data even though only nearly 2D sections are analyzed. Note that this distinguishes stereology from “planimetric” microscopic image analysis of pixels. Thus, for V (dimension 3), the appropriate test system consists of points (dimension 0). For S (dimension 2), the appropriate test system consists of lines (dimension 1). For L (dimension 1), the appropriate test system consists of planes (dimension 2). Finally, for N (dimension 0), the appropriate test system consists of volumes (dimension 3).

**Table 4:** Relationship of structural quantities and stereological principles

Parameter (dimension)	Appearance in 2D section (dimension)	Probe (dimension)	Event	Measurement	Density estimate
Volume *V* (3)	Area *A* (2)	Point *P*_*T*_ (0)	Point lies in volume (“hits” *A*)	Point count *P(x)*	*V*_*V*_*(x)* = *P(x)/P*_*T*_
Surface area *S* (2)	Boundary *B* (1)	Line *L*_*T*_ (1)	Line intersects surface (“hits” *B*)	Intersection count *I(x)*	*S*_*V*_*(x)* = *2·I(x)/L*_*T*_
Length *L* (1)	Point *Q* (0)	Plane *A*_*T*_ (2)	Plane transects line (“hits” *Q*)	Transect count *Q(x)*	*L*_*V*_*(x)* = *2·Q(x)/A*_*T*_
Number *N* (0)	–	Volume (Disector) *A*_*T*_*·t* (3)	Disector volume “hits” particle top	Top count *Q*^*¯*^*(x)*	*N*_*V*_*(x)* = *Q*^*¯*^*(x)/A*_*T*_*·t*

Basic structural quantities that can be estimated (and their dimension), their appearance in single thin microscopic sections (and their dimension), the appropriate geometric probes to measure them (and their dimension), the events generated by the interaction of the probe with the structure, the counts (measurements) that result, and the formulae for calculation of densities in the reference volume. These densities (ratios) per unit reference volume have to be converted to total values by multiplying them with the total volume of the reference space (see Fig. 1). After [37]

This simple relationship has important consequences. It assures not only the 3D nature of stereological data. It also tells you that single thin microscopic sections cannot be used to obtain unbiased estimates of particle number in 3D space. This information is simply lost due to sectioning—a fact that you have to live with, although you may find publications where this fact is ignored (a typical case of the “dimension trap”, see Step 1 above). You may argue that you see particles in single thin microscopic sections, so why not just count them? What you actually see is only profiles of particles, created by the sectioning process. However, the number of (sectioned) particle profiles per 2D area is not proportional to the number of particles in 3D space. Instead, you may regard particle profiles in a 2D section as transects in the section plane. Thus, the number of particle profiles (or transects) per area is proportional to their length (or height perpendicular to the section plane) in 3D—not their number (compare length estimation in Fig. 4 and Table 4). In other words, sectioned particle profiles in a thin microscopic section are a biased sample of particles, with larger particles more likely to be sectioned and therefore over-represented—clearly a violation of the equal opportunity sampling principle.

What is then the solution for particle number estimation? It is termed the *disector* [44]. This concept, which is probably unfamiliar to you, requires some explanation. In its original description, it consists of two sections from one tissue block (hence the name: di-sector). This creates test volumes defined by the area of unbiased counting frames [11] placed over corresponding fields of view on the two sections, multiplied by the distance between the two sections (in the common case of consecutive sections the section thickness). This is the *physical* *disector*. Regarding the effort to produce section pairs and corresponding fields of view, an efficient alternative may be to focus through one thick section in z direction (*optical* *disector*). However, the typical applications of the disector for lung research are based on physical disectors because the counting events are easier to define [34]. The counting events in physical disectors follow the principle *“now* *you* *see* *it,* *now* *you* *don’t”*. This means that the particle is present in one section (termed the sampling section for counting), but not the other (termed the look-up section for comparison). In other words, the disector volume contains the top of particles as counting events, irrespective of their size. Alternatively, the appearance/disappearance of a well-defined characteristic of that particle, e.g. the nucleolus in the case of cells, can be used. In that sense, the disector counting rule is the 3D extension of the 2D counting rule provided by the unbiased counting frame (see Fig. 6).

The disector is the one stereological tool for unbiased estimation of particle number in microscopy. It is also the tool for unbiased (i.e. size-independent equal opportunity) sampling of particles for further measurements, e.g. their mean volume (reviewed in [27]). This applies to any kind of “particle” in the broadest sense, even if they have incomplete boundaries and connections, such as lung alveoli. Here, the alveolar opening rings are used for counting (Fig. 6) [21, 38].

So far, so good. But there is also bad news: In stereology, the counts have to be done manually. As yet, no automated image analysis system is (artificially) intelligent enough to recognize and measure complex biological structures with a certainty and efficiency as good as a human expert doing quick and simple point and intersection counts. While this may change sometime in the future, the principles of unbiasedness in sampling that are provided by stereology will still apply then. Neither are accuracy and precision of data assured by automated image analysis (see Step 6 below) nor is the “dimension trap” addressed by “planimetry” of pixels (see Step 1 above).

Don’t forget that these counts on microscopic sections only provide you with ratios (Table 4). You have to convert them to total values by multiplying them with the total volume of the reference space (see Steps 1 and 3 above and Fig. 1). The principles of stereological test systems and applications to lung disease models are illustrated in [30, 37] and [6]. A neat free online software tool to create digital test systems, termed the STEPanizer, is described in [45]. A further option is the combination of a slide scanner with commercial stereology and image analysis software (whole slide stereology) (as used in [43]).

### Step 6: How many counts are enough?

A correct but unsatisfactory answer to this question could be “it depends on the particular conditions of your study”. Giving a more helpful answer requires the introduction of the terms accuracy and precision. Keep in mind that, due to the inherent stochastic nature of the approach, all stereological data are estimates. *Accuracy* refers to the validity or unbiasedness of the data (“Is this estimate the true value?”). Bias refers to systematic error, i.e. systematic deviation of the data from the true value. Potential sources of bias include tissue deformation during sample preparation for microscopy (e.g. paraffin embedding, as discussed above under Step 2), improper sampling (violating the equal opportunity rule, see Step 4 above), geometric model assumptions that deviate from reality (e.g. “alveoli are spherical”), and incomplete visualization (in the end, you can only measure what you see). In contrast, *precision* refers to the reproducibility of the data (“Do independent repetitions produce estimates of the same value?”). It mainly depends on the sampling design, in particular the size and distribution of the sample.

In your study, you have to differentiate between accuracy and precision. They both characterize the quality of your data, but in a very different way. Bias is a dangerous enemy, because it cannot be detected in your final data (you don’t know the deviation of your data from the true value because you don’t know the true value—otherwise you wouldn’t have to do the stereological study to estimate it), nor can it be decreased by working harder, i.e. by increasing the number of measurements per individual. You don’t know what you are fighting against. So the only solution is to avoid bias in the first place by using appropriate (i.e. unbiased) methods. In contrast, the precision of the data can easily be controlled because it can be calculated from the data and, if necessary, increased by including more samples and measurements. In other words, you just have to work a little harder (increase the number of measurements per individual) to obtain data with higher precision.

But still, how much is enough? This question can be dealt with at various levels of complexity. There is a basic rule of thumb (that works in almost all practical cases) which recommends to count between 100 and 200 events (“hits”) per study object for the estimation of a given parameter of interest. Of course these 100–200 counting events should be well distributed over the whole lung (or your target subcompartment within the lung) by using a smart sampling design (as outlined in Step 4 above and exemplified below). When this condition is met, the *coefficient* *of* *error* *(CE)* introduced by the estimation procedure (a mathematical expression of the imprecision of your data) is almost always considerably smaller than the biological variation between individuals. Then, the overall observed *coefficient* *of* *variation* *(CV)* of a study group is dominated by the biological variation between individuals (the “signal”) and not by the CE of the stereological estimate (the “noise”). As an example, if you use 5 tissue blocks sampled from a mouse lung by SURS and about 10 fields of view selected by SURS from one section per tissue block each, you only need about 3 counting events on your structure of interest per field of view to reach about 150 counting events in that lung. Once you have sampled the fields of view properly, the actual counting per lung is probably done in less than 1 h. You can adjust this sampling design to your particular needs at the beginning in the little pilot study you will have to do anyway (see Step 1 above). It does not make sense to count more than that in order to decrease the CE further. Your goal should be to count enough to reach a sufficient precision, but not more than that. Don’t waste your time generating unnecessary over-precision (and then even demonstrating your ignorance by proudly stating that you counted over 10,000 test points per lung). Smart data are more important than big data. In summary, be as accurate as possible, but only as precise as necessary.

You are not satisfied with this rule of thumb? There are more sophisticated ways of checking whether the precision of your estimate is sufficient in the context of your study. The CE of stereological estimates can be predicted and calculated based on mathematical models (discussed in the references below). Then, you can formally verify to what extent the CE contributes to the CV of a study group (this CV can easily be computed as the standard deviation divided by the mean of that group). Aim at CE^2^/CV^2^ between 0.2 and 0.5. Values below indicate that you have counted too much, values above that you have to count more. The theoretical aspects of variance estimation in stereology (a field of active research for mathematicians) are covered in [2]. Formulae for CE calculation and prediction of various estimators are given in [19].

In case you need to increase the precision (or decrease the CE) of your estimate, where should you invest the additional effort? It is by far more efficient to include more individuals per study group (to address inter-individual variability) and more tissue blocks per lung (to address intra-individual variability) than to increase the number of test points or lines per field of view. The principle of putting more effort into the higher stages of the sampling sequence is known as *“Do* *more* *less* *well!”* ([15], after a quote by Ewald Weibel).

### Step 7: Reporting your methods and results

Your next study will include nice stereological data according to ATS/ERS standards which increases the likelihood that the prestigious journal that rejected your last submission is now willing to publish this one. What should you report in your paper? In the methods section, be specific about the way you fixed the lungs (Step 2), determined their volumes (Step 3), selected (Step 4) and processed (Step 2) the samples. Which parameters were estimated using which test system (Step 5)? How much did you count per parameter and lung, and how were those counts distributed over the lung (Step 6)?

In the results section, any stereological data should be preceded by a qualitative description of the microscopic findings (Step 1). Always report total values, i.e. data per whole lung (Steps 1 and 3). Depending on the context of your study, it may be interesting to provide ratios in addition (e.g. when you want to compare data normalized per unit tissue volume), but never forget that these ratios “per µm^3^ of lung tissue” alone cannot be interpreted as totals (don’t fall into the “reference trap”). For some purposes, the inverse of ratios might be of particular interest. While N per V is subject to the “reference trap”, V per N equals mean particle volume. While S per V is subject to the “reference trap”, V per S equals mean barrier thickness (Step 1).

In case you want to illustrate your stereological data as graphs, use scatter plots (1 dot = 1 individual) [29]. For different experimental groups, report the mean and, as a measure of the variability of the individual measurements in a group, the standard deviation (SD). Do not use the standard error of the mean (SEM) instead of the SD, because this is statistically incorrect (see [10]) and often abused as “error bar cosmetics”. Instead of the SD, consider reporting the CV (a normalized SD that is dimensionless, which allows comparisons of data variations between different parameters), perhaps even the CE (if you have calculated it) (Step 6). Trust us, reviewers with experience in lung stereology will like that.

## Further information

It is far beyond the scope of this short primer to cover all aspects of stereology in depth. But perhaps it can function as an appetizer. How to get further information? For further reading, excellent textbooks ([2] for statisticians, [19] for biologists and physicians) and review articles [4, 7, 9, 16, 17, 24, 25] on general principles and applications of stereology are available. Various aspects of practical applications of design-based stereology in respiratory research are described in several reviews [6, 30, 31, 34, 35, 37, 41, 48]. Stereology as the method of choice for quantitative assessment of lung structure has been published as an official research policy statement of the ATS and the ERS [20]. The combination of lung stereology and advanced 3D imaging methods has been reviewed recently [22]. Maybe you even become interested in the history of stereology [8, 47] and some of the pioneers of biomedical stereology like Ewald Weibel [26, 36] and Hans Jørgen Gundersen [3, 46].

You still want to know more about stereology? Our recommendation is to attend a course on practical stereology. These courses, usually between 3 and 5 days, are offered regularly at various places all over the world. Many of them are announced via the International Society for Stereology and Image Analysis. With a mixture of lectures and exercises, sometimes even lab practicals, they provide an excellent introduction into the theory and practice of biomedical stereology and, in addition, direct interactions with expert stereologists and thus the opportunity to discuss your specific project with them. These expert stereologists are happy to help further and accept visitors in their labs for teaching them the nuts and bolts. When we got started in lung stereology we also went though this sequence: reading—course—lab visit [3], so we have good reasons to recommend it.

## Conclusions

Stereology offers a versatile toolbox to obtain valid quantitative data on lung structure. It is approved by the ATS and the ERS. It offers a solid mathematical foundation and transparency of its algorithms. It offers built-in unbiasedness and tailored precision. It offers elegance and good scientific practice. And it is fun. What more can you ask for?

## Data Availability

Not applicable.

## Abbreviations

1D: One-dimensional
2D: Two-dimensional
3D: Three-dimensional
ATS: American Thoracic Society
CE: Coefficient of error
CV: Coefficient of variation
ERS: European Respiratory Society
IUR: Isotropic uniform random
L: Length
N: Number
S: Surface area
SD: Standard deviation
SEM: Standard error of the mean
SURS: Systematic uniform random sampling
$\overline{\tau }$: Mean barrier thickness
V: Volume
$\overline{\nu }$: Mean particle volume
VUR: Vertical uniform random
