# Correction to: A short primer on lung stereology

**DOI:** 10.1186/s12931-022-01936-8

**Published:** 2022-03-08

**Authors:** Matthias Ochs, Julia Schipke

**Affiliations:** 1 Institute of Functional Anatomy, Charité-Universitätsmedizin Berlin, Corporate Member of Freie Universität Berlin and Humboldt Universität Zu Berlin, Philippstr. 11, 10115 Berlin, Germany; 2 German Center for Lung Research (DZL), Berlin, Germany; 3 Institute of Functional and Applied Anatomy, Hannover Medical School, Hannover, Germany; 4 Biomedical Research in Endstage and Obstructive Lung Disease, Member of the German Center for Lung Research (DZL), Hannover, Germany

## Correction to: Ochs and Schipke Respiratory Research (2021) 22:305 10.1186/s12931-021-01899-2

Following publication of the original article [1], the authors identified errors in Table 3, Figure 1 and few headings style. It have been included in this correction.

The original article has been corrected.

1. Table 3 layout has been updated and given below

**Table 3:** The 7 crucial steps of a stereological study of the lung

Step	Points to consider
1. Planning your stereological study	Pilot study Target compartment Stereological parameters Sampling and analysis design
2. Preparation of lung tissue	Route of fixation Composition of fixative Post-fixation and processing Dehydration and embedding
3. Definition and measurement of the reference space	Fixed lung volume: - Archimedes principle - Cavalieri estimator
4. Sampling	Randomization of location: - Systematic uniform random sampling - Cascade sampling - Stratified sampling - Fractionator sampling Randomization of spatial orientation - Isotropic uniform random sections - Vertical sections
5. Doing the measurements	Dimension of stereological parameter of interest Dimension of geometric probe (test system) Disector for number estimation
6. How many counts are enough?	Accuracy Precision Coefficient of error
7. Reporting your methods and results	Total values Ratios and inverse of ratios Scatter plots Standard deviation

2. Formula in Figure 1 legend has been updated.

The total volume of type II cells, *V*(type II), is then obtained as

$$V\left( {type\, II} \right) = V\left( {lung} \right)\cdot {V_{V} \left( {par/lung} \right)} \cdot {V_{V} \left( {alvsep/par} \right)} \cdot {V_{V} \left( {type II/alvsep} \right)}$$

3. Heading level has been updated with the Capital letters

- *Step 1: Planning your stereological study*
- *Step 2: Preparation of lung tissue*
- *Step 3: Definition and measurement of the reference space*
